# Comprehensive Geriatric Assessment and Clinical Outcomes in the Older People at the Emergency Department

**DOI:** 10.3390/ijerph18116164

**Published:** 2021-06-07

**Authors:** Cheng-Fu Lin, Po-Chen Lin, Sung-Yuan Hu, Yu-Tse Tsan, Wei-Kai Liao, Shih-Yi Lin, Tzu-Chieh Lin

**Affiliations:** 1 Department of Emergency Medicine, Taichung Veterans General Hospital, Taichung 40705, Taiwan; chengfue@gmail.com (C.-F.L.); darren.lynne@gmail.com (P.-C.L.); song9168@pie.com.tw (S.-Y.H.); janyuhjer@gmail.com (Y.-T.T.); kents90124@hotmail.com (W.-K.L.); 2 Center for Geriatrics & Gerontology, Taichung Veterans General Hospital, Taichung 40705, Taiwan; 3 Institute of Clinical Medicine, School of Medicine, National Yang Ming Chiao Tung University, Taipei 11221, Taiwan; 4 Division of Endocrinology and Metabolism, Department of Internal Medicine, Taichung Veterans General Hospital, Taichung 40705, Taiwan

**Keywords:** older people, acute care, emergency department, geriatric assessment, frailty

## Abstract

Visits by older people to the Emergency Department (ED) have increased in recent decades with higher revisiting and admission rates after discharge, particularly for those with frailties. This study used a before–after design aimed at evaluating Comprehensive Geriatric Assessment (CGA) screening in older ED patients (aged ≥ 75 years) during the 12-month preintervention period. Additionally, a CGA-based structured follow-up program after ED discharge was executed during the next 12-month intervention period. Amongst the 358 participants (median age 82 years), involving 122 in the preintervention period and 236 in the intervention period, 77 participants (21.5%) were identified as pre-frailty, while 274 (76.5%) were identified as frail using the Fried frailty phenotype. One-hundred ten (110) (30.7%) patients revisited the ED with 73 (20.4%) being admitted and 20 (5.6%) dying within three months after ED discharge. Compared with preintervention and intervention period, it was shown that the rates of admission at the index ED visit (50.8% vs. 23.1%), and mortality (10.7% vs. 3.0%), were both were significantly reduced. Using multivariate regression analysis, it was shown frailty was significantly associated with three-month mortality after adjusting for potential confounders. On the contrary, the program significantly decreased admission and death rate. It is suggested that frailty was prevalent amongst the older ED patients, and should be screened for in order to decrease revisits/admissions after ED discharge.

## 1. Introduction

Older people visiting the Emergency Department (ED) often have more chronic diseases, and are characterized by certain vulnerable features such as the presence of geriatric syndromes, including cognitive impairment, delirium, mood disorder, polypharmacy, frailty, falls, incontinence, and potentially atypical disease presentation [1]. Accordingly, they will have higher rates of healthcare and support services utilization [2], particularly involving more frequent ED visits [3,4,5,6,7]. Older people who visit the ED often suffer from more complicated cognition and physical problems; hence, they may have longer lengths of stay, a higher admission probability, and the requirement for more resources to help improve their condition [8]. However, in the ED, the medical staff faces more pressure in the context of time-based targets, ED flow, and resource allocation, all of which is made even more demanding due to an overcrowded and busy environment, thus, further compounding the problems for the older people visiting the ED [9]. All these conditions undoubtedly bring challenges surrounding the proper management of older people in the ED, where upholding the quality and safety of care in older people is essential.

Older people are usually more likely to present themselves to the ED as the department provides a convenient point of entry into the healthcare system [10]. However, traditional ED management is disease-oriented, causing responses to be particularly limited for frail older people suffering from multimorbidity. It has been reported that up to 60% of the older people who visit the ED are frail and have higher ED utilization rates, longer lengths of stay, an increased likelihood of admission and greater mortality [10]. Additionally, frailty often interplays with multiple comorbidities, functional decline, cognitive disorder, polypharmacy, and caretaker strain all of which can be predisposed to worse outcomes and greater care needs [11]. Therefore, more time spent on both comprehensive assessment and management by multidisciplinary care teams is necessary in older people with frailty in order to improve their outcomes [11]. In 2014, the Geriatric Emergency Department Guidelines were published with the aim of offering geriatric-friendly ED care by focusing on staffing, administration, physical environment, and leadership [12]. A recent review reported on the partial effects that ED intervention strategies such as discharge planning and case management have on clinical and utilization outcomes for older adults [13].

In Taiwan, there has been a progressive increase in ED utilization by older people, although any evidence that explored the characteristics of frailty in older people in the ED and its effect on outcomes was less studied in this population. Information taken from the Comprehensive Geriatric Assessment (CGA) guide regarding an individual’s health and social care needs has shown to prevent the inappropriate admission of older patients who were capable of returning immediately to their own homes [14]. This study aimed to examine the relevant factors associated with clinical outcomes, including post ED discharge revisit/admission rates and mortality, in older people visiting EDs. Through use of a before–after study design, we evaluated the effectiveness of CGA screening in older ED patients during the preintervention period, while during the intervention period, a CGA-based structured follow-up program after ED discharge was executed.

## 2. Methods

### 2.1. Study Design

**Figure 1:**
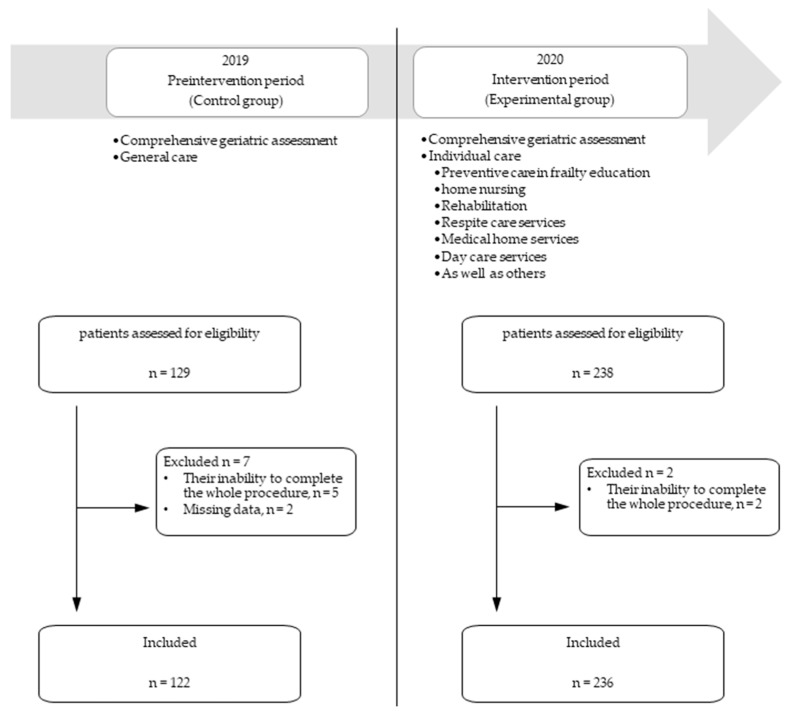
Overview of the implementation process and data collection periods.

This was a prospective before–after study performed in a medical center in Taiwan (Figure 1). The study design included a preintervention period (January 2019 to December 2019), and an intervention period (January 2020 to December 2020). During the preintervention period, training courses consisted of lectures and staff education on how to implement the CGA. During the intervention period, the caring program was commenced according to the results of the CGA. The study was approved by the Institutional Review Board (IRB) of Taichung Veterans General Hospital (IRB No: CE18256A). All methods were carried out in accordance with relevant guidelines and regulations.

### 2.2. Participants

This study targeted Chinese-speaking, community-dwelling ED patients aged 75 years or older who had one of the following diseases: chronic heart failure, chronic kidney disease, diabetes mellitus, or chronic obstructive pulmonary disease in a stable clinical condition. Exclusion criteria included any unstable or terminal disease and/or the inability to cooperate in a long-term follow-up assessment.

### 2.3. Preintervention Period

The Acute Care for Elders (ACE) model was led by an emergency medicine specialist and a geriatrician. The well-trained case manager conducted CGA amongst the participants to identify their geriatric syndromes within 12 h after ED visiting. The parameters of CGA included the patients’ demographic information, including age, gender, body mass index (kg/m^2^), education level, marital status, decision-making individual, caregiving support, and measurement data. The measurement data involved cognitive impairment (defined as scores <24 on the Chinese version of the Mini-Mental State Examination, MMSE [15]), mood disorder (defined by scores ≥2 on the 5-item Chinese Geriatric Depression Scale, GDS-5 [16]), medical condition (defined by the Charlson comorbidity index, CCI [17]), polypharmacy (defined as currently using >4 drugs [18]), malnutrition (defined by scores <12 on the Mini-Nutritional Assessment-Short Form, MNA-SF [19]), physical function (assessed by the Barthel index of Activities of Daily Living, ADL [20] and the Lawton Instrumental Activities of Daily Living Scale, IADL [21]), health-related quality of life (measured by the Chinese version of the EQ-5D system) [22], as well as frailty in accordance with Fried’s definition of the frailty phenotype [23], which was evaluated based upon the presence of three or more criteria: weight loss, low physical activity, exhaustion, weakness (hand grip strength), and slowness (walking speed). Weight loss meant an unintentional decrease in body weight more than 5 kg-weight within one year. Low physical activity meant a weighted score of kilocalories expended per week based on each self-report activities less than 383 kcal/week for male and 270 kcal/week for female. Exhaustion meant self-report fatigue. Weakness meant hand grip strength less than 26 kg for male and 18 kg for female. Slowness meant walking speed slower than 0.8 m/s. Frailty was defined as the presence of three or more of these criteria, prefrailty was defined as the presence of one or two of these criteria, and robust was defined as the absence of any of these criteria.

### 2.4. Intervention Period

The caring program consisted of case meetings involving the creation of individualized care plans according to the CGA. This may have had several interventions, including preventive care in frailty education, home nursing, rehabilitation and respite care services, medical home services, and day care services, as well as others.

### 2.5. Outcome

At one month and three months after ED discharge, we followed up the patients over the telephone to inquire about their physical performance, healthcare, and support services utilization. Additionally, revisits to the ED, admission for hospitalization (at or after the index ED visit), and mortality within three months were also recorded.

### 2.6. Statistical Analyses

Continuous variables were expressed as median and Interquartile Range (IQR, 25–75%). Categorical data were expressed as both number and percentage. The significance of the difference between groups was assessed using the Mann–Whitney U test or Kruskal–Wallis test for continuous variables and the Chi-Square test for categorical variables. The influence of one or more confounding factors in dichotomous outcomes was evaluated by deriving univariate and multivariate logistic regression models. Statistical analyses were performed using SPSS version 22.0 (SPSS Inc., Chicago, IL, USA). A two-tailed *p*-value of <0.05 was considered statistically significant.

## 3. Results

During the recruitment period, a total of 367 older patients aged ≥75 years were reviewed for eligibility, with nine patients being excluded due to their inability to complete the whole procedure. This resulted in 358 patients being enrolled, 122 (34.1%) in the preintervention period and 236 (65.9%) in the intervention period. Between the preintervention and intervention groups, the patients within the intervention group had more comorbidities, better cognitive function, and better nutritional status (Table 1). For all the subjects involved, the median age was 82.0 years (IQR: 79.0–87.0 years) with a female-to-male ratio of 57:43. One hundred sixty-six (46.4%) patients possessed decision making skills, while 208 (58.1%) required assistance from a caregiver. Chronic illness evaluation was through CCI 2 (1–4) and polypharmacy was evident in 231 (64.5%) patients. The CGA of the MMSE score was 22 (18–26), ADL was 90 (68.8–100), IADL was 5 (2–7), MNA-SF was 11 (9–12), and frailty was 3 (3–4). Among the five frailty components, decreased hand grip strength, walking speed, and self-reported physical activities were most commonly seen in more than 80% of older patients.

Between participants being classified as either frail, prefrail, or robust, 77 participants (21.5%) were identified as pre-frailty, while 274 (76.5%) were identified as frail (Table 2). The level of frailty increased significantly with age and a decreased handgrip strength. Additionally, the patients with frailty were less educated, and less able to take care of or make decisions for themselves. Finally, frail older patients experienced a higher percentage of comorbid diseases, disabilities, malnutrition, depressive symptoms, cognitive impairment, and poor life quality than those who were in the robust classification. The clinical outcomes showed that older patients with frailty had an increased three-month mortality rate (Table 2).

Regarding all the patients, 117 (32.7%) were subsequently admitted after their ED visit, 110 (30.7%) revisited the ED, 73 (20.4%) were admitted to the hospital, and 20 (5.6%) died during the three-month follow-up period. Compared with those in the preintervention group, for those in the after caring program it was shown that the rates of admission at the index ED visit (23.1% vs. 50.8%) and mortality (3.0% vs. 10.7%) were both significantly reduced. Besides, there was also a decreased trend of ED revisiting (35.3% vs. 28.4%), and admission rates (23.0% vs. 19.1%) within three months after their index ED visit, though no statistical significance. Using regression analysis, it was shown that caring program, male gender, and MNA-SF scores were associated with decreased odds ratios of admission following their index ED visit, and after adjustment for the other covariates, the caring program remained significant (Table 3). Cognitive impairment was associated with increased odds ratios for those patients revisiting the ED within three months, although caring for oneself and making one’s own decisions decreased this risk (Table 4). Cognitive impairment and frailty were risk factors regarding three-month admission; however, decision making on one’s own decreased this risk (Table 5). In terms of mortality, it was found that decision making on one’s own and MNA-SF scores, along with participation in the caring program were all associated with decreased odds ratios in three-month mortality, although frailty was associated with an increased mortality risk in univariate analysis. After adjustment, the association between frailty, the caring program, and mortality remained significant (Table 6).

## 4. Discussion

In this study, we found a high prevalence of geriatric syndromes in older people visiting the ED, where 76.5% of patients had frailty, 64.8% had nutrition issues, 64.5% had polypharmacy, and 48.4% had cognitive impairment as determined by the CGA. Additionally, those patients with frailty, a physical disability, cognitive impairment, malnutrition risk, and depressive symptoms had higher chances for revisiting the ED, admission, and/or mortality. Through implementation of the CGA-based structured caring program at the ED, it was discovered that the rates of three-month ED revisit and admission after the index ED visit were borderline decreased, with hospitalization at the index ED visit, as well as three-month mortality after ED discharge significantly reduced.

There are many models surrounding ED-based care, which have been discussed for older people visiting the ED because of it being an unfriendly setting for them. Several studies have shown the effects of different models of ED-based care, however, the optimal one remains controversial and still needs to be quantified [8,13]. ACE, a model for interdisciplinary care in association with CGA, has been developed to both promote quality of care and improve clinical outcomes in inpatient settings [24,25]. However, although the CGA process is time-consuming, and may not be suitable for the busy ED environment, several recent studies have shown that specialized geriatrician-led teams within EDs that perform CGA can prevent inappropriate admission of older patients after ED discharge [14]. The ACE design incorporates a friendly environment for older people, as well as a multidimensional team that works together to identify the vulnerability of older people using CGA. That particular team evaluates frail older patients by checking their medical, psychosocial and functional capabilities in order to develop a coordinated and integrated plan for treatment, follow-up, and prevention of disability [26]. Furthermore, performing CGA in the ED can improve outcomes in issues such as admission, return revisits, and death [11,14]. In line with those reports, we found that after the implementation of the ACE model in the ED, the rate of three-month revisiting and admission, along with the mortality rate, was reduced. To accomplish this integrative care model in EDs, nurse-led discharge [27] and ED care coordination models have been proposed [28]. Furthermore, implementation of the screening program regarding geriatric syndromes during routine ED care does not negatively impact the ED process [29], but rather increases the compliance of the older patients to maintain follow-up. In our study, the ED care model was supported by external geriatricians and case managers who conducted CGA amongst the older ED visitors. However, this process requires additional manpower, is time-consuming, and not naturally suited to the busy ED environment. Further studies for the purpose of evaluating the effectiveness of both CGA and care planning, as well as the coordination between ED staff and the other specialists, are still required in order to supply commissioners and other stake holders with evidence of their value [14].

Associated factors for frailty, such as biological, behavioral, social, and medical aspects, are reported to be different in both genders [30]. Thus, sex-specific strategies for prevention and treatment of frailty have been suggested. For example, exercise programs appear to be effective for both sexes, while men seem to benefit more from nutritional interventions [31]. In our study, although there were different female proportions between pre- and intervention period, it was shown that a frailty caring program was helpful for patient’s outcome (i.e., admission and mortality) independent of genders. Further research of multiple component strategies with consideration of issues relevant to each sex may be necessary.

It is well known that frailty is a state of vulnerability and increases the risk of disability, admission, institutionalization, and death [32]. In this study, we found that 76.5% of the patients who underwent the CGA were classified as frail. Frailty prevalence in ED patients has been examined in numerous studies, and ranges from approximately 7% to 80%, depending upon the characteristics of the patients, and the tools used to assess frailty [33]. Some studies have used established frailty tools, such as a frailty phenotype, a frailty index, or the clinical frailty scale; whereas other studies have used tools developed for other purposes, such as the ISAR screening tool or the Vulnerable Elder Survey, to screen for frailty [33]. In a previous study, it was shown that by using the Fried frailty phenotype, 55.4% of older people at EDs were classified as prefrail, and 30.4% as frail, numbers that appear to be similar to our observation [34], whilst another research study in the US reported that the frailty prevalence was 20% [35]. It is proposed that the frailty status of older patients is a dynamic process with frequent transitions in short periods in which they become more or less frail [36,37]. When patients are acutely unwell, which can become exhausting for older patients, that may exacerbate measured objective parameters such as hand grip strength and gait speed, reflective of a limitation imposed by the patient’s acute illness or injury. On the contrary, those who would initially be assessed as frail based for instance on a slow walking speed according to one of the CHS criteria, may become less frail with a remarkable recovery after a short period of adequate therapy. In our study, frailty was assessed within 12 h after ED admission rather than at the time after overnight stay or at discharge from ED in some studies [34,35]. In the individual component measures of frailty, slowness was the most frequently followed by weakness and self-reported decreased physical activity. Therefore, the higher frailty proportion in our patients may possibly represent a “roof” of frailty prevalence in older ED patients.

Current ED triage depends upon the severity of illness, and may frequently result in undertriage for older patients who are experiencing other associated complex problems, such as functional decline, frailty, and polypharmacy, as well as a lack of family care and social support, as shown in this study. All these geriatric disorders interact with clinical illness, and can add to prognosis risks [33,38]. Our study has shown that frailty predicted ED revisiting, admission, and mortality. This finding, as well as previous reports, implicate that the ED provider should not only manage acute medical illness, but also recognize any associated problems and contributing factors in older patients who are frail. Consequently, a care plan that can be specifically addressed for the care needs of an older patient lessens the risk of further disability, hospitalization, and mortality [39]. Several studies have reported that after the identification of frailty in older people seen in the ED, the provision of community-based services after discharge may improve their outcomes [40]. In our study, an intervention service program involving life modification, preventive care in the community, physical therapy at home, personal medication review, and home visits by a physician and nurse were all shown to be beneficial when discussing clinical outcomes. Overall, those previous findings, as well as ours, support the effectiveness of both frailty screening and an intervention program at the ED as two ways to reduce the burden of ED revisits and/or admission. However, it should be noted that in a busy ED environment where there is no geriatric specialist, development of a proper and feasible tool is still necessary in order to highlight both pre- and post-discharge support. Future research comparing the effectiveness of rapid frailty screening versus comprehensive geriatric assessment is still needed.

Our study found that adequate self-care ability and decision making both play important roles surrounding a patient’s clinical outcome, including revisiting the ED, admission, and mortality. A healthy patient may benefit from their ability to make their own decisions, while also offering relief to his/her caregiver and, thus, lessening their burden. These findings are consistent with a previous study that revealed unplanned admission was based upon a combination of factors including oneself, the family, and the physician [41]. Secondly, nearly half of the patients visiting EDs were experiencing cognitive impairment, with one third displaying symptoms of depression. Both cognitive impairment and depression are common in older people visiting EDs, which in turn may result in complex interplay surrounding outcomes [42]. As cognitive impairment and depression may be linked to treatment adherence and self-management, ED revisiting, admission, and mortality may increase after older people are discharged from the ED [43,44,45]. Third, nutritional status is also a major issue in older people, with malnutrition usually underdiagnosed [1], and the prevalence of malnutrition being high in frailty patients [46]. Malnutrition not only reduces functional capacity but also increases complications such as frailty related mechanical falls, delayed wound healing, hospital admission, and mortality [47,48]. In our study, we found that both cognitive function and nutritional status independently affected clinical outcomes in older people visiting the ED.

This study has some limitations. First, patients were not randomized using a before and after design, which, therefore, may not provide a causality between the intervention and the outcomes. Additionally, the preintervention patients who had CGA performed may have taken part in other services at other hospitals, so there may have been some contamination of this group. However, the main outcomes were affected by time period, and through this we could not detect substantial differences in the two groups. Second, we did not examine the effects of specific interventions in the service programs with regards to the participants’ outcomes. It is, therefore, possible that some of the activities may not have been optimal for all of the older people in the study, thus, making it necessary to develop a future program that can be tailored to the individual needs of each older person. Third, the study nurses did not manage to include all of the older patients consecutively due to the unpredictable nature of patient flow in the ED, possibly leading to selection bias. Fourth, our study population was comprised of patients aged 75 years and older who were presented to the ED of a hospital with a nonsurgical illness, hence, limiting any generalizability to different clinical settings, surgical illnesses, or younger age groups. Finally, this study was performed in one hospital with admission data having been collected in that same hospital; thus, we cannot exclude the possibility that some patients could have been admitted elsewhere.

## 5. Conclusions

This study shows that geriatric syndromes such as frailty were frequently seen amongst older people visiting the ED, and were associated with admission, and mortality, all of which were improved by an ED caring program. It is suggested that CGA be integrated within EDs for these older people, with the multidisciplinary intervention subsequently improving outcomes.

## Figures and Tables

**Table 1:** Baseline characteristics of the participants.

	Total (n = 358)		Preintervention (n = 122)		Intervention (n = 236)		*p* Value
**Demographic characteristics**							
Age (years)	82	(79–87)	82	(79–89)	82	(78–86)	0.083
Gender							0.001
Female	204	(57.0%)	85	(69.7%)	119	(50.4%)	
Male	154	(43.0%)	37	(30.3%)	117	(49.6%)	
BMI (kg/m^2^)	24.1	(21.4–26.6)	24.20	(21.8–27.3)	23.91	(21.3–26.2)	0.284
Educational level							0.222
Illiterate	79	(22.1%)	20	(16.4%)	59	(25.0%)	
Literate	22	(6.2%)	8	(6.6%)	14	(5.9%)	
Primary school	146	(40.8%)	58	(47.5%)	88	(37.3%)	
Junior high school	31	(8.7%)	11	(9.0%)	20	(8.5%)	
Senior high school	42	(11.7%)	16	(13.1%)	26	(11.0%)	
University	38	(10.6%)	9	(7.4%)	29	(12.3%)	
**Geriatric assessment characteristics**							
Falls in the last month	58	(16.4%)	24	(20.5%)	34	(14.4%)	0.192
Falls in the last year	111	(31.4%)	38	(32.5%)	73	(30.9%)	0.863
Decision-making person—oneself ^a^	166	(46.4%)	60	(49.2%)	106	(44.9%)	0.512
Caregiver—oneself ^b^	150	(41.9%)	48	(39.3%)	102	(43.2%)	0.554
Charlson comorbidity index	2	(1–4)	2	(1–3)	2	(1–4)	0.021
Polypharmacy	231	(64.5%)	78	(63.9%)	153	(64.8%)	0.959
Mini-mental state examination	22	(18–26)	21	(16–25)	23	(19–27)	0.014
Barthel index before emergency department ^c^	90	(68.8–100)	90	(55–100)	90	(70–100)	0.536
Lawton scale ^d^	5	(2–7)	5	(2–7)	5	(2–7)	0.393
Mini Nutritional Assessment Short-Form	11	(9–12)	9	(7–11)	11	(9–13)	<0.001
Frailty components	3	(3–4)	3	(3–4)	3	(2.3–4)	0.075
Exhaustion ^e^	159	(44.4%)	63	(51.6%)	96	(40.7%)	0.048
Weight loss ^f^	48	(13.4%)	26	(21.3%)	22	(9.3%)	0.002
Hand grip strength ^g^	291	(81.3%)	104	(85.3%)	187	(79.2%)	0.167
Walking speed ^h^	309	(86.3%)	96	(78.7%)	213	(90.3%)	0.003
Low energy expenditure ^i^	303	(84.6%)	107	(87.7%)	196	(83.1%)	0.247
EQ-5D utility index ^j^	0.5	(0.2–0.7)	0.5	(0.2–0.7)	0.5	(0.3–0.7)	0.460
EQ- visual analogue scale	60	(45–70)	50	(30–60)	60	(50–78.8)	<0.001

Continuous data were expressed median (IQR), Categorical data were expressed in number and percentage. ^a^ Decision-making person was divided into oneself and other. ^b^ Caregiver was divided into oneself and other. ^c^ Activities of Daily Living Scale. ^d^ Instrumental Activities of Daily Living Scale. ^e^ Self-report fatigue. ^f^ Weight loss 5 kg-weight within one year. ^g^ Male less than 26 or female less than 18 kg. ^h^ Less than 0.8 m/s. ^i^ Self-report physical activity and need for help. ^j^ Health-related quality of life.

**Table 2:** Comparison between frailty, pre-frailty, and non-frailty.

	Robust (n = 7)		Pre-Frail (n = 77)		Frail (n = 274)		*p* Value
**Demographic characteristics**							
Age (years)	77	(77–83)	80	(78–84.5)	83	(79–88)	0.001
Gender							0.148
Female	6	(85.7%)	48	(62.3%)	150	(54.7%)	
Male	1	(14.3%)	29	(37.7%)	124	(45.3%)	
BMI (kg/m^2^)	24.9	(23.8–27.7)	24.3	(22.0–27.2)	24.0	(21.3–26.4)	0.503
Educational level							0.003
Illiterate	0	(0%)	9	(11.7%)	70	(25.6%)	
Literate	0	(0%)	0	(0%)	22	(8.0%)	
Primary school	4	(57.1%)	35	(45.5%)	107	(39.1%)	
Junior high school	0	(0%)	12	(15.6%)	19	(6.9%)	
Senior high school	1	(14.3%)	8	(10.4%)	33	(12.0%)	
University	2	(28.6%)	13	(16.9%)	23	(8.4%)	
**Geriatric assessment characteristics**							
Falls in the last month	1	(14.3%)	3	(4.0%)	54	(20.0%)	0.004
Falls in the last year	2	(28.6%)	9	(11.8%)	100	(37.0%)	<0.001
Decision-making person—oneself	7	(100%)	49	(63.6%)	110	(40.2%)	<0.001
Caregiver—oneself	5	(71.4%)	54	(70.1%)	91	(33.2%)	<0.001
Charlson comorbidity index	0	(0–2)	2	(1–3)	2	(1–4)	<0.001
Polypharmacy	2	(28.6%)	37	(48.1%)	192	(70.1%)	<0.001
Mini-mental state examination	27.5	(24.8–28.5)	26	(23–27)	21	(16–25)	<0.001
Barthel index before emergency department	100	(100–100)	100	(95–100)	80	(53.8–95)	<0.001
Lawton scale	7	(6–8)	7	(6–8)	4	(1–6)	<0.001
Mini Nutritional Assessment Short-Form	12	(11–13)	12	(11–13.5)	10	(8–12)	<0.001
EQ-5D utility index	0.8	(0.6–0.8)	0.7	(0.7–0.8)	0.4	(0.1–0.7)	<0.001
EQ- visual analogue scale	80	(65–89.5)	60	(50–70)	60	(30–70)	0.009

Continuous data were expressed median (IQR). Categorical data were expressed in number and percentage.

**Table 3:** Predictors of admission at the index emergency department visit.

	Simple Model				Multiple Model (n = 318)			
	OR	(95%CI)		*p* Value	OR	(95%CI)		*p* Value
**Demographic characteristics**								
Age (years)	1.04	(0.99–1.08)		0.100				
Gender								
Female	ref.				ref.			
Male	0.58	(0.37–0.92)		0.019	0.80	(0.46–1.40)		0.433
BMI (kg/m^2^)	0.99	(0.95–1.04)		0.805				
Educational level								
Illiterate	ref.				ref.			
Literate	1.27	(0.43–3.73)		0.662	1.01	(0.28–3.61)		0.992
Primary school	1.87	(1.00–3.50)		0.049	1.44	(0.63–3.28)		0.390
Junior high school	2.45	(1.01–5.94)		0.048	1.89	(0.64–5.59)		0.248
Senior high school	1.69	(0.74–3.88)		0.213	1.11	(0.39–3.18)		0.840
University	1.98	(0.85–4.59)		0.113	2.46	(0.90–6.71)		0.079
**Geriatric assessment characteristics**								
Falls in the last month	0.95	(0.52–1.74)		0.862				
Falls in the last year	0.76	(0.46–1.24)		0.266				
Decision-making person—oneself	0.69	(0.44–1.08)		0.102				
Caregiver—oneself	0.81	(0.52–1.27)		0.359				
Charlson comorbidity index	0.95	(0.84–1.08)		0.448				
Polypharmacy	0.83	(0.52–1.30)		0.411				
Mini-mental state examination	0.99	(0.95–1.03)		0.646				
Barthel index before emergency department	0.99	(0.99–1.00)		0.193				
Lawton scale	0.96	(0.88–1.04)		0.278				
Mini Nutritional Assessment Short-Form	0.86	(0.79–0.94)		0.001	1.03	(0.91–1.18)		0.604
Frailty	1.20	(0.97–1.50)		0.100				
EQ-5D utility index	0.72	(0.39–1.32)		0.287				
EQ- visual analogue scale	1.00	(0.98–1.01)		0.460				
**Caring program**								
Preintervention	ref.				ref.			
Intervention	0.29	(0.18–0.47)		<0.001	0.33	(0.18–0.58)		<0.001

**Table 4:** Predictors of emergency department revisit within three months.

	Simple Model				Multiple Model			
	OR	(95%CI)		*p* Value	OR	(95%CI)		*p* Value
**Demographic characteristics**								
Age (years)	0.99	(0.95–1.04)		0.786				
Gender								
Female	ref.							
Male	0.84	(0.53–1.32)		0.443				
BMI (kg/m^2^)	1.00	(0.95–1.05)		0.881				
Educational level								
Illiterate	ref.							
Literate	1.39	(0.51–3.76)		0.515				
Primary school	0.98	(0.54–1.80)		0.956				
Junior high school	1.54	(0.64–3.67)		0.333				
Senior high school	1.22	(0.54–2.72)		0.632				
University	0.99	(0.42–2.33)		0.985				
**Geriatric assessment characteristics**								
Falls in the last month	0.91	(0.49–1.70)		0.777				
Falls in the last year	1.33	(0.82–2.15)		0.242				
Decision-making person—oneself	0.58	(0.37–0.93)		0.022	0.64	(0.33–1.25)		0.190
Caregiver—oneself	0.57	(0.36–0.92)		0.020	0.93	(0.48–1.79)		0.831
Charlson comorbidity index	1.06	(0.94–1.20)		0.346				
Polypharmacy	1.19	(0.74–1.92)		0.470				
Mini-mental state examination	0.95	(0.91–0.99)		0.007	0.97	(0.92–1.01)		0.137
Barthel index before emergency department	0.99	(0.99–1.00)		0.215				
Lawton scale	0.97	(0.89–1.05)		0.473				
Mini Nutritional Assessment Short-Form	0.92	(0.84–1.00)		0.063				
Frailty	1.19	(0.95–1.49)		0.126				
EQ-5D utility index	0.80	(0.43–1.49)		0.483				
EQ- visual analogue scale	1.00	(0.99–1.01)		0.707				
**Caring program**								
Preintervention	ref.							
Intervention	0.73	(0.46–1.16)		0.183				

**Table 5:** Predictors of admission within three months after index emergency department visit.

	Simple Model				Multiple Model			
	OR	(95%CI)		*p* Value	OR	(95%CI)		*p* Value
**Demographic characteristics**								
Age (years)	0.97	(0.92–1.02)		0.230				
Gender								
Female	ref.							
Male	0.79	(0.46–1.33)		0.368				
BMI (kg/m^2^)	0.99	(0.94–1.05)		0.846				
Educational level								
Illiterate	ref.							
Literate	1.25	(0.40–3.94)		0.697				
Primary school	1.25	(0.63–2.47)		0.528				
Junior high school	1.02	(0.36–2.94)		0.965				
Senior high school	0.71	(0.25–1.99)		0.517				
University	1.14	(0.44–2.98)		0.792				
**Geriatric assessment characteristics**								
Falls in the last month	0.58	(0.26–1.28)		0.176				
Falls in the last year	0.87	(0.50–1.54)		0.641				
Decision-making person—oneself	0.46	(0.26–0.79)		0.005	0.63	(0.34–1.17)		0.143
Caregiver—oneself	0.77	(0.46–1.31)		0.341				
Charlson comorbidity index	1.17	(1.02–1.35)		0.025	1.12	(0.96–1.30)		0.138
Polypharmacy	1.25	(0.72–2.16)		0.428				
Mini-mental state examination	0.93	(0.89–0.98)		0.00 *	0.96	(0.91–1.01)		0.114
Barthel index before emergency department	1.00	(0.99–1.01)		0.463				
Lawton scale	0.97	(0.89–1.07)		0.569				
Mini Nutritional Assessment Short-Form	0.94	(0.85–1.04)		0.253				
Frailty	1.33	(1.02–1.74)		0.037	1.13	(0.81–1.57)		0.463
EQ-5D utility index	0.89	(0.44–1.80)		0.736				
EQ- visual analogue scale	1.00	(0.98–1.01)		0.788				
**Caring program**								
Preintervention	ref.							
Intervention	0.79	(0.46–1.35)		0.388				

**Table 6:** Predictors of death within three months after index ED visit.

	Simple Model				Multiple Model			
	OR	(95%CI)		*p* Value	OR	(95%CI)		*p* Value
**Demographic characteristics**								
Age (years)	0.99	(0.91–1.08)		0.883				
Gender								
Female	ref.							
Male	1.09	(0.44–2.70)		0.854				
BMI (kg/m^2^)	1.00	(0.91–1.10)		0.979				
Educational level								
Illiterate	ref.							
Literate	0.00	(0.00–		0.998				
Primary school	0.68	(0.24–1.89)		0.455				
Junior high school	0.71	(0.14–3.62)		0.680				
Senior high school	0.25	(0.03–2.11)		0.203				
University	0.28	(0.03–2.35)		0.239				
**Geriatric assessment characteristics**								
Falls in the last month	1.76	(0.61–5.05)		0.293				
Falls in the last year	1.19	(0.46–3.06)		0.725				
Decision-making person—oneself	0.27	(0.09–0.83)		0.022	0.28	(0.07–1.12)		0.072
Caregiver—oneself	0.58	(0.22–1.54)		0.272				
Charlson comorbidity index	1.10	(0.87–1.40)		0.413				
Polypharmacy	1.69	(0.60–4.78)		0.319				
Mini-mental state examination	0.93	(0.86–1.00)		0.065				
Barthel index before emergency department	0.99	(0.98–1.01)		0.205				
Lawton scale	0.93	(0.79–1.09)		0.355				
Mini Nutritional Assessment Short-Form	0.79	(0.67–0.93)		0.005	0.96	(0.71–1.30)		0.781
Frailty	2.09	(1.21–3.60)		0.008	2.48	(1.07–5.74)		0.033
EQ-5D utility index	0.54	(0.16–1.86)		0.329				
EQ- visual analogue scale	0.97	(0.95–0.99)		0.007	0.99	(0.96–1.01)		0.260
**Caring program**								
Preintervention	ref.				ref.			
Intervention	0.26	(0.10–0.66)		0.005	0.21	(0.05–0.95)		0.043

## Data Availability

Not applicable.

## Abbreviations

ACE: Acute Care for Elders; ADL: activities of daily living; CCI: Charlson comorbidity index; CGA: comprehensive geriatric assessment; ED: emergency department; GDS-5: five-item Geriatric Depression Scale; IADL: Instrumental Activities of Daily Living; MMSE: mini-mental state examination; MNA-SF: Mini-Nutritional Assessment-Short Form.
